# Introduction to ultrafast meets ultrasmall: exploring the uncharted territory of quantum dynamics

**DOI:** 10.1039/d6na90007k

**Published:** 2026-02-24

**Authors:** Kristina R. Rusimova, Thomas Siday, Marcello Righetto

**Affiliations:** a Department of Physics, University of Bath UK; b Centre for Photonics and Photonic Materials, University of Bath UK; c University of Birmingham UK; d University of Padova Italy

## Abstract

Kristina R. Rusimova, Tom Siday and Marcello Righetto introduce the *Nanoscale Advances* themed collection titled ultrafast meets ultrasmall: exploring the uncharted territory of quantum dynamics.
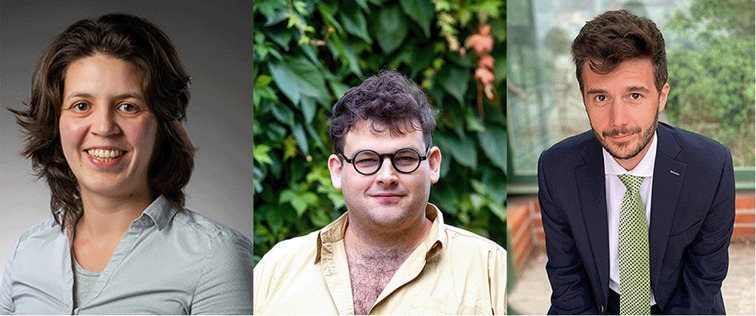

Physical processes in nanoscale and quantum systems unfold over astonishingly short distances – atoms, molecules, and atomic defect sites in two-dimensional materials – and on equally astonishingly short timescales, from femtoseconds to picoseconds and beyond. As researchers push opto-electronic and quantum technologies toward the atomic and molecular scale, the critical challenge becomes not only to observe flows of charge, spin, energy, and light, but to control them with unprecedented spatial and temporal precision. In this context, the convergence of ultrafast optics and time-resolved scanning probe microscopies (SPM), such as scanning tunnelling microscopy (STM) and atomic force microscopy (AFM), has opened a powerful frontier.

In recent years, major advances have emerged from groups bridging the far-field ultrafast community with the atomic-scale SPM community. Techniques now allow pump-probe experiments with tip-localised excitation, coherent spin manipulation of single atoms, and real-time control at the single-molecule level. This themed collection of *Nanoscale Advances*, “Ultrafast meets ultrasmall: exploring the uncharted territory of quantum dynamics”, presents a curated set of these breakthroughs, highlighting how spatial precision and temporal resolution are being combined to interrogate and manipulate quantum materials in previously inaccessible regimes.

The themed collection begins with contributions that illustrate the control of quantum spins at the atomic scale. Choi *et al.* (https://doi.org/10.1039/D5NA00316D) review how electron spin resonance integrated into STM enables single-spin addressability and coherent control, including the implementation of multi-qubit gates on identical on-surface qubits, establishing ESR-STM as a platform for atomic-scale quantum circuits. Building on this, Switzer *et al.* (https://doi.org/10.1039/D5NA00421G) provide a numerical demonstration of entanglement generation in two titanium adatoms using ESR-STM with microwave pulse sequences. They achieve a Bell state fidelity of ≈93%, illustrating the potential for on-surface quantum logic and atom-based quantum circuits. Together, these studies exemplify how ultrafast spin control can be harnessed at the ultimate spatial limit.

Complementary contributions explore charge and energy dynamics in molecular and nanoscale systems. Sufyan *et al.* (https://doi.org/10.1039/D5NA00727E) investigate electron transfer in noble-gas endofullerenes on Pb/Cu(111) using tunnelling spectroscopy and DFT, revealing complex intramolecular and substrate coupling, highlighting the subtle interplay of structure and ultrafast dynamics in molecular systems. Luferau *et al.* (https://doi.org/10.1039/D5NA00307E) apply scattering-type near-field optical spectroscopy to GaAs/InGaAs core–shell nanowires, mapping ultrafast carrier re-combination from a few picoseconds up to 100 ps and showing the roles of bimolecular and surface-assisted processes in controlling nanoscale carrier dynamics.

Plasmonic and coherent light-matter interactions at the nanoscale are also explored. Toffoletti and Collini (https://doi.org/10.1039/D4NA00917G) use two-dimensional electronic spectroscopy to track the ultrafast dephasing of localized surface plasmons in gold nanorods, directly measuring dephasing times of ∼8–13 fs and demonstrating how shape and resonance energy govern coherent dynamics; insights critical for plasmonic and quantum nanophotonic applications.

The issue further highlights atomic-scale defects and interfacial charge transfer in two-dimensional and organic systems. Casado *et al.* (https://doi.org/10.1039/D5NA00501A) probe defects in MoTe_2_ monolayers using STM/STS, showing how tip-induced band bending reveals charge states of individual defects, linking structure to electronic behavior. Schaal *et al.* (https://doi.org/10.1039/D4NA00462K) examine PTCDA molecules on h-BN/Ni(111), observing spontaneous integer charge transfer and radical anion formation, connecting molecular-scale charge redistribution to interfacial energy-level alignment.

Finally, the issue encompasses ultrafast carrier dynamics in two-dimensional materials. Sharma *et al.* (https://doi.org/10.1039/D2NA00678B) investigate hot-carrier cooling in hydrogen-intercalated bilayer graphene using femtosecond optical-pump/THz-probe spectroscopy, finding picosecond-scale relaxation dominated by electron–optical phonon scattering, illustrating how substrate decoupling can dramatically alter ultrafast relaxation pathways.

Taken together, this themed collection demonstrates a unifying theme: the convergence of ultrafast temporal resolution with atomic- and molecular-scale spatial precision is now enabling researchers to interrogate and manipulate the fundamental quantum processes that govern materials, molecular assemblies, and low-dimensional systems. By bridging SPM, ultrafast optics, and theoretical analysis, these contributions chart a path toward fully controlling quantum dynamics in nanoscale systems, opening opportunities for next-generation optoelectronic, plasmonic, and quantum devices.

